# Malaria care-seeking among febrile patients in Myanmar and Thailand: an accelerated failure time analysis

**DOI:** 10.21203/rs.3.rs-7373958/v1

**Published:** 2025-09-09

**Authors:** Khaing Zin Zin Htwe, Pyae Linn Aung, Piyarat Sripoorote, Myat Thu Soe, Pattamaporn Petchvijit, Poh Poh Aung, Amnat Khamsiriwatchara, Saranath Lawpoolsri, Liwang Cui, Daniel M Parker, Myat Phone Kyaw, Jetsumon Sattabongkot, Jaranit Kaewkungwal

**Affiliations:** Mahidol University; Mahidol University; Mahidol University; Myanmar Health Network Organization; Mahidol University; Mahidol University; Mahidol University; Mahidol University; University of South Florida; University of California, Irvine; Myanmar Health Network Organization; Mahidol University; Mahidol University

**Keywords:** malaria, care-seeking time, accelerated failure time model, Greater Mekong Subregion, febrile illness

## Abstract

**Background:**

Timely malaria care-seeking and treatment is crucial to prevent severe illness and reduce onward transmission. This study assessed the time to care-seeking and identified its associated factors among febrile patients attending community-based malaria facilities in Myanmar and Thailand.

**Methods:**

Longitudinal data were collected from febrile patients suspected of malaria who sought care through Village Health Volunteers in Myanmar (December 2017–June 2021) and at malaria clinics and posts in Thailand (January 2018–June 2024). Standardized case record forms in local languages were used at diagnosis. Descriptive statistics and log-logistic Accelerated Failure Time (AFT) models were used to estimate Time Ratios (TRs).

**Results:**

In Myanmar (n = 2,960), the mean fever duration at diagnosis was 1.8 days (SD: 1.3). Longer time to care-seeking was associated with Shan ethnicity (TR: 1.48; 95% CI: 1.41–1.55), other ethnicities (TR: 1.24; 95% CI: 1.08–1.41), farmers (TR: 1.12; 95% CI: 1.0–1.25), uncertain malaria history (TR: 1.26; 95% CI: 1.09–1.47), and diagnosis with *Plasmodium falciparum* (TR: 1.10; 95% CI: 1.02–1.19) or *P. vivax* (TR: 1.23; 95% CI: 1.13–1.34). Shorter delays were associated with daily bed net use and diagnosis during the third or fourth quarters of the year. In Thailand (n = 15,576), the mean fever duration was 2.9 days (SD: 1.8). Longer delays were linked to farmers (TR: 1.07; 95% CI: 1.02–1.11), pre- or primary education (TR: 1.02; 95% CI: 1.00–1.04), uncertain malaria history (TR: 1.10; 95% CI: 1.06–1.14), diagnosis with *P. falciparum* (TR: 1.27; 95% CI: 1.09–1.46), *P. vivax* (TR: 1.20; 95% CI: 1.17–1.23), or other malaria species (TR: 1.32; 95% CI: 1.12–1.56), and diagnosis during the third (TR: 1.03; 95% CI: 1.00–1.05) or fourth quarters of the year (TR: 1.06; 95% CI: 1.04–1.09). Shorter care-seeking times were observed among non-agricultural occupations such as merchants and monks, individuals with prior malaria episodes, and occasional or daily bed net users.

**Conclusions:**

Delays in malaria care-seeking remain common in both Myanmar and Thailand. The identified risk factors, including ethnicity, occupation, malaria history, and preventive behaviors, should be considered in the design of targeted interventions to promote timely care-seeking in malaria-endemic settings.

## Background

Myanmar and Thailand remain among the last malaria-endemic countries in the Greater Mekong Subregion (GMS), a region committed to eliminating all malaria by 2030 (1). Historically, Myanmar accounted for the majority of malaria cases in the region. Intensive control measures led to an 82% drop in reported cases in Myanmar, from 481,204 in 2012 to 85,019 in 2017 (2). However, recent political instability and weakening health services reversed this trend dramatically. According to the World Health Organization (WHO), Myanmar’s annual case count jumped from approximately 78,000 in 2019 to nearly 847,000 in 2023 (3). In Thailand, although the malaria burden is significantly lower and elimination targets had nearly been achieved by 2020, case numbers also increased to 16,675 in 2023, up from 10,153 in 2022. About 42% of these cases were classified as imported, and this rise has been the most pronounced in the border provinces, reflecting cross-border transmission from Myanmar (4).

In Myanmar, key interventions to control malaria have included the widespread distribution of long-lasting insecticidal nets (LLINs), deployment of integrated community malaria volunteers (ICMVs) in endemic villages, and the expansion of case detection and treatment (5–7). Village health volunteers (VHVs), trained and equipped with rapid diagnostic tests (RDTs) and artemisinin-based combination therapy (ACT), offer frontline malaria diagnosis and treatment (8, 9). These community-focused interventions led to significant reductions in malaria cases until disruptions to the health system began in 2021 (10). Similarly, Thailand has leveraged an extensive network of Malaria Clinics (MCs), VHVs, and Migrant Health Volunteers (MHVs), which also provide expanded access to diagnosis and treatment in border and forest-fringe communities (11). Active case detection and the distribution of LLINs are prioritized in high-risk areas (12). Both countries have implemented robust surveillance and response strategies, most notably the ‘1-3-7’ case notification system, whereby malaria cases are meant to be reported within 1 day, investigated within 3 days, and targeted interventions initiated within 7 days (13). Despite their pronounced implementation, the effectiveness of these strategies remains suboptimal.

Timely care-seeking by febrile patients is critical for malaria control and elimination. WHO emphasizes that every case of malaria should be diagnosed and treated promptly, ideally within 24–48 hours of symptom onset, to clear infections before they produce infectious gametocytes (14). Prompt diagnosis and effective treatment not only shorten the duration of illness and normally prevent the development of severe disease but also directly interrupt the transmission cycle by reducing the human parasite reservoir. This effect may be more immediate in *P. vivax* infections, where gametocytes appear early and are frequently present (15, 16). Delay or failure to seek care can leave these infections persist for weeks, continuing to infect mosquitoes and sustaining local transmission (17). Such delays are particularly concerning in areas with partial immunity or predominant *P. vivax* infections, which can present with delayed or relapsing symptoms that may not prompt immediate care (18). Studies in these settings have reported late care-seeking behavior, particularly among migrant and rural populations. For example, Myanmar migrant workers in Thailand exhibit delayed malaria care-seeking behavior, increasing risks of severe illness and sustained onward transmission (19). Understanding determinants of care-seeking delays can help guide interventions. A previous study in Myanmar indicated that lower education levels and limited malaria knowledge contributed to delays in diagnosis and treatment (20). By understanding where and why febrile patients delay or forego diagnosis, malaria programs can adapt strategies to ensure timely testing and treatment, thus reducing the infectious reservoir and moving from control to elimination (21, 22).

Despite intensive control efforts, cross-border malaria remains a dynamic challenge in the GMS (23). Myanmar and Thailand share epidemiological foci, but national health systems and local contexts differ (24). Few studies have directly compared patient care-seeking across these neighboring countries. Insights into differences and similarities in health-seeking behaviors and factors associated with delayed care between the two settings can inform tailored improvements in surveillance, diagnostics, and community engagement (25). Considering this, the present study measured reported malaria care-seeking time and identified its associated factors among febrile patients attending community-based malaria services in Myanmar and Thailand.

## Methods

### Study Design and Setting

**Figure 1:**
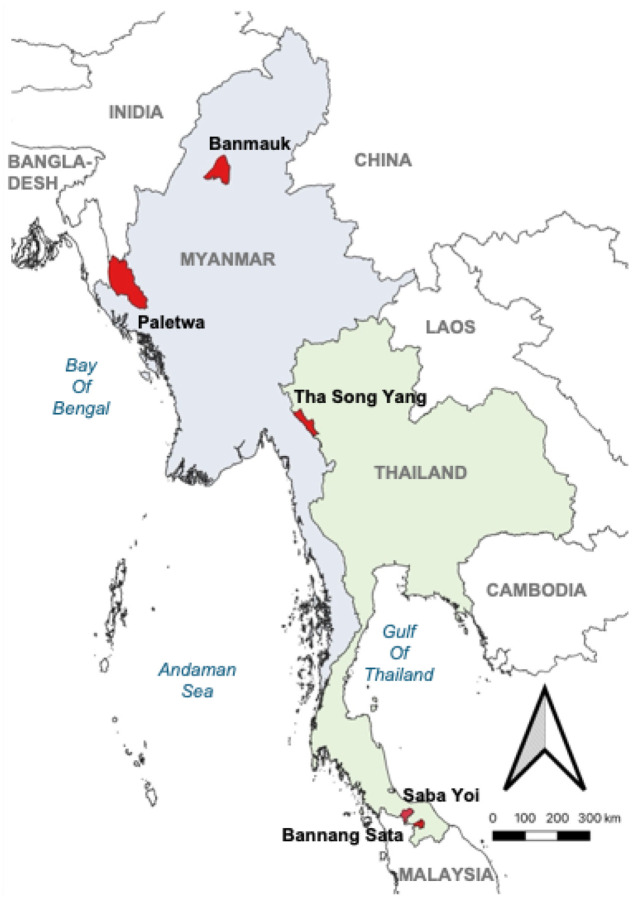
Map of study sites in Thailand and Myanmar. The map was created using QGIS (version 3.34.2-Prizren). Administrative boundaries and geographic features were adapted from publicly available shapefiles (https://gadm.org/)

This study employed a cross-sectional design conducted in selected malaria-endemic areas of Myanmar and Thailand (Fig. 1).

In Myanmar, two townships from regions reporting the highest malaria incidence, Banmauk Township in Sagaing Region and Paletwa Township in Chin State, were purposively selected. Within each township, 8–10 villages with high reported malaria cases and accessible transportation routes were chosen in consultation with local health authorities and administrative personnel. Each village had one to two VHVs, who received initial malaria diagnosis and treatment training (3–5 days), followed by annual refresher courses. Equipped with RDTs and anti-malarial drugs, VHVs provided free malaria diagnostic and treatment services to all individuals seeking care.

In Thailand, Tha Song Yang District in Tak Province, located along the southeastern Myanmar border, was purposively selected as a high-malaria-endemic site. Additionally, Bannang Sata and Saba Yoi Districts in southern Thailand were included to represent areas of residual transmission along the Malaysian border. From these three districts, two to three malaria clinics and posts per site participated, offering free malaria diagnosis and treatment to all individuals, regardless of citizenship or migration status (documented or undocumented).

### Data collection

Data were collected using standardized case record forms (CRFs), developed collaboratively by the research teams from both countries. Initially prepared in English, CRFs were translated and back-translated into Burmese and Thai to ensure accuracy.

All frontline healthcare personnel and VHVs involved in data collection received comprehensive training covering good clinical practice, CRF item clarification, informed consent procedures, and reporting protocols. CRFs captured sociodemographic data (age, sex, ethnicity, occupation, education), malaria history within the past 12 months, bed net usage, and self-reported fever duration. The main study outcome, malaria care-seeking time, was calculated via self-reported fever duration (days of fever before presentation) and confirmed through objective measurement using infrared forehead thermometers. A febrile case was defined as either self-reported current fever or measured temperature ≥ 38°C at diagnosis.

Patients presenting with malaria-like symptoms were tested using RDTs (combo *Pf*/*Pv*) in Myanmar and microscopy in Thailand. While awaiting test results, CRFs were completed by VHVs (Myanmar) and clinic staff (Thailand). Completed CRFs were scanned and securely uploaded to a cloud-based data platform hosted by the Faculty of Tropical Medicine, Mahidol University, Thailand. Data were regularly reviewed for accuracy and completeness using standardized quality-control protocols.

### Study samples and data handling

The final dataset was extracted by the research team in early 2025. The data covered the period from December 2017 to June 2021 in Myanmar, where field activities ceased due to political instability, and from January 2018 to June 2024 in Thailand. All individuals presenting with suspected malaria were initially eligible for inclusion. A few records were excluded if key variables, particularly fever duration, were missing, or if the individual neither reported nor presented with fever. For repeated visits by the same individual, only the first visit was included in the analysis. After exclusions, the final analytic sample comprised 2,960 individuals from Myanmar and 15,576 from Thailand.

## Statistical Analysis

Fever duration until diagnosis was summarized using both mean (standard deviation) and median (range) for each study site in Myanmar and Thailand. To visualize the time to malaria care seeking, Kaplan–Meier survival curves were generated and stratified by country and malaria species. These non-parametric curves provide an overview of the empirical distribution of fever duration until diagnosis without assuming any specific statistical distribution. Accelerated Failure Time (AFT) models were explored to identify the best-fitting model for the data. Among the candidate distributions (exponential, Weibull, log-normal, log-logistic, and Gaussian), the log-logistic AFT model showed the best fit based on Akaike Information Criterion (AIC), Bayesian Information Criterion (BIC), and diagnostic plots: quantile–quantile (Q–Q) and residual plots.

Sociodemographic and clinical characteristics were summarized with frequencies and percentages. Categorical variables included age group (< 5, 5–14, 15–34, and ≥ 35 years), sex (male, female), ethnicity (Chin, Rakhine, Shan, and Other for Myanmar; Karen, Thai, and Other for Thailand), occupation (agriculture/farmer, child/student, mining applicable to Myanmar only, unemployed, and other), education level (no formal education, pre/primary school, secondary school, and university or higher), malaria history within the past 12 months (no, yes, and do not remember), bed net usage (not at all, some days, and every day), malaria diagnosis (negative, *P. falciparum*, *P. vivax*, and other malaria), and diagnosis quarter (Q1: January-March, Q2: April-June, Q3: July-September, and Q4: October-December). The “Other” category under ethnicity included Kachin, Mon, Myanmar, and other minority groups. For occupation, “Other” included merchants, monks, and other roles. In the “Other malaria” category, cases in Myanmar consisted of mixed infections with *P. falciparum* and *P. vivax*, while cases in Thailand also included mixed infections, *P. knowlesi*, *P. malariae*, and *P. ovale* infections. These were all categorized under “Other malaria” due to their low frequency relative to standalone *P. falciparum* and *P. vivax* infections. Malaria diagnosis by microscopy in Thailand was confirmed by expert re-examination. The results were obtained from the expert examination.

To determine factors associated with fever duration, separate log-logistic AFT models for each country were fitted to estimate time ratios (TRs), 95% confidence intervals (CIs), and *p*-values. Variance inflation factor (VIF) values below 2 indicated no concerning multicollinearity among predictors. All statistical analyses were conducted using RStudio (version 2024.12.1 + 563).

## Results

A total of 2,960 patients from Myanmar and 15,576 from Thailand were included in the analysis. Overall, the mean fever duration at diagnosis was shorter in Myanmar (1.8 days; SD: 1.3) compared to Thailand (2.9 days; SD: 1.8). Median fever durations were 1 day (range: 1–14) for Myanmar and 2 days (range: 1–45) for Thailand. Within Myanmar, patients from Banmauk reported a longer average fever duration (2.1 days; SD: 1.1) than those from Paletwa (1.7 days; SD: 1.4). In Thailand, Saba Yoi recorded the longest mean fever duration (4.6 days; SD: 2.5), followed by Bannang Sata (3.2 days; SD: 1.6) and Tha Song Yang (2.8 days; SD: 1.7) (Table 1).

**Figure 2:**
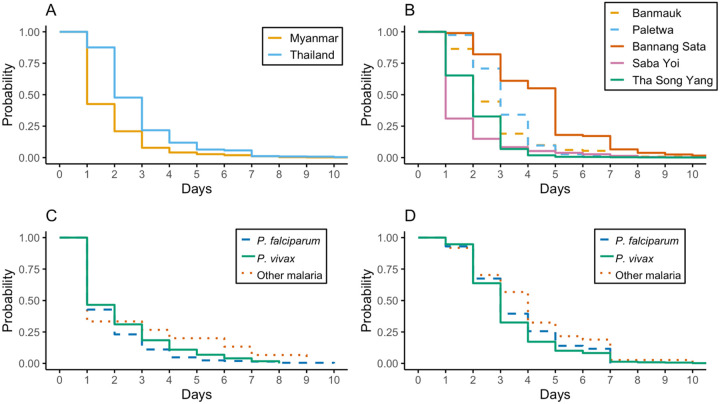
Kaplan-Meier survival curves for malaria care-seeking time. Probability of not seeking care over time (truncated at 10 days) by (A) country, (B) study site, (C) malaria species in Myanmar, and (D) malaria species in Thailand

Figure 2 displays Kaplan–Meier curves comparing the time from fever onset to malaria care-seeking across countries, study sites, and infecting species. In Panel A, the curve for Myanmar lies to the left of that for Thailand, indicating shorter delays among Myanmar patients. Panel B shows site-level comparisons: Saba Yoi and Bannang Sata (Thailand) exhibit earlier drops, suggesting faster care-seeking among some patients despite their higher mean/median durations. In Panels C and D, species-stratified curves show that in Myanmar, patients with *P. falciparum* sought care fastest, followed by *P. vivax* and other species; in Thailand, the fastest care-seeking occurred among *P. vivax* cases, followed by *P. falciparum*, while those with other species experienced the longest delays.

Factors influencing malaria care-seeking delays were analyzed separately for Myanmar (Table 2) and Thailand (Table 3).

In Myanmar, participants of Shan ethnicity experienced a 48% longer delay in care-seeking compared to those of Chin ethnicity (TR: 1.48; 95% CI: 1.41–1.55), and those from other ethnic groups also experienced longer delays (TR: 1.24; 95% CI: 1.08–1.41). Participants who could not recall whether they had malaria in the past 12 months had a 26% longer time to care-seeking compared to those without recent malaria (TR: 1.26; 95% CI: 1.09–1.47). Daily bed net users sought care 7% faster than non-users (TR: 0.93; 95% CI: 0.88–0.99). Compared to malaria-negative individuals, those diagnosed with *P. falciparum* and *P. vivax* took 10% (TR: 1.10; 95% CI: 1.02–1.19) and 23% longer (TR: 1.23; 95% CI: 1.13–1.34), respectively, to seek care. Agricultural workers also had longer delays compared to the unemployed (TR: 1.12; 95% CI: 1.00–1.25). Mining occupation was marginally associated with longer care-seeking delays (TR: 1.15; 95% CI: 1.00–1.34), with the confidence interval bordering on statistical significance. Additionally, participants diagnosed during Q3 and Q4 were significantly quicker to seek care than those diagnosed in Q1 (TR: 0.91; 95% CI: 0.86–0.96 and TR: 0.92; 95% CI: 0.87–0.98, respectively). No statistically significant associations were found for age, sex, or education level (Table 2).

In Thailand, ethnicity showed no significant effect; however, occupation and education significantly influenced care-seeking. Agricultural workers sought care 7% slower than the unemployed (TR: 1.07; 95% CI: 1.02–1.11), while those in other occupations (e.g., merchants, monks) sought care 6% faster (TR: 0.94; 95% CI: 0.90–0.98). Participants with pre-primary or primary education experienced slightly longer delays than those with no formal education (TR: 1.02; 95% CI: 1.00–1.04). A history of malaria in the past 12 months was associated with a faster response, with an 11% shorter time to care-seeking (TR: 0.89; 95% CI: 0.86–0.93), whereas those who could not recall their malaria history had 10% longer delays (TR: 1.10; 95% CI: 1.06–1.14). Bed net use significantly reduced delays: occasional users sought care 11% faster (TR: 0.89; 95% CI: 0.86–0.92), and daily users were 17% faster (TR: 0.83; 95% CI: 0.80–0.85) compared to non-users. Diagnosis with *P. falciparum*, *P. vivax*, and other malaria species significantly increased care-seeking delays by 27% (TR: 1.27; 95% CI: 1.09–1.46), 20% (TR: 1.20; 95% CI: 1.17–1.23), and 32% (TR: 1.32; 95% CI: 1.12–1.56), respectively, relative to malaria-negative individuals. Additionally, participants diagnosed in Q3 and Q4 had significantly longer delays than those diagnosed in Q1 (TR: 1.03; 95% CI: 1.00–1.05 and TR: 1.06; 95% CI: 1.04–1.09, respectively). No significant differences in time to care-seeking were observed across age groups and sex (Table 3).

## Discussion

Malaria remains a significant health threat in the GMS, where early diagnosis and treatment are crucial to reduce morbidity, mortality, and transmission (14). This study, based on real-world care-seeking delays reported by febrile patients attending malaria facilities in Myanmar and Thailand, provides valuable insights into the factors influencing timely malaria care-seeking that may inform malaria control efforts.

Overall, this study observed longer delays in Thailand than in Myanmar. Several contextual factors may explain this disparity. In Thailand, border areas host substantial populations of migrant workers and displaced persons, many of whom face barriers to timely care (26, 27). These include fear of legal repercussions due to undocumented status (24), language and cultural differences, limited awareness of available services, and concerns about income loss or job insecurity if time is taken off to seek treatment (28–30). In contrast, patients in Banmauk and Paletwa, Myanmar, are primarily indigenous Burmese ethnic groups, often residing in settings where community-based malaria services are more accessible and familiar (31). It is, however, important to note that the data collection in Myanmar concluded in June 2021, prior to deterioration in the security situation. Therefore, the shorter delays observed in Myanmar might not reflect the current context.

Geographic and structural health system barriers may further contribute to delayed care, particularly in Thailand’s forested, mountainous, and conflict-affected regions. Long travel distances, difficult terrain, and insecurity may limit timely access to clinics, particularly in southern Thai districts like Bannang Sata and Saba Yoi (32–34). Conversely, areas such as Tha Song Yang, with established non-governmental organizations (NGOs) and refugee health services, demonstrated shorter delays (35). Within Myanmar, patients in Banmauk Township, Sagaing region, had longer delays than those in Paletwa, Chin State. Banmauk is a forested area with limited roads and has seen rising malaria incidence, potentially hindering rapid access (36). These intracountry variations highlight how local infrastructure, security, and service accessibility influence care-seeking behaviors.

Ethnic disparities were also evident: patients identifying as Shan or other ethnicities sought care significantly later than Chin patients in Myanmar. Although all participants were recruited from Banmauk (Sagaing Region) or Paletwa (Chin State), Banmauk is inhabited by ethnically diverse populations. Shan communities in Banmauk might experience disrupted access due to cultural and linguistic barriers contributing to care-seeking delay. Interestingly, Chin participants, despite coming from a remote and impoverished area, sought care more promptly. One plausible explanation is that Chin State has been the focus of intensified malaria control activities, including NGO support and community health volunteer programs, particularly due to high prevalence (37, 38). These findings suggest that culturally sensitive and linguistically appropriate health promotion interventions may help improve timely care-seeking in diverse settings like Banmauk.

In both countries, occupation was a significant determinant of delay. Compared to unemployed individuals, agricultural workers sought care more slowly in Myanmar and Thailand, whereas Thai participants in other occupations, such as merchants, monks, sought care faster. Farming livelihoods often involve long workdays and seasonal cycles; farmers may delay or avoid leaving the fields for treatment, and clinics may be far from farms. Consistent with our findings, a study in Indonesia found that rural farmers had a poorer understanding of prompt malaria treatment than public-sector workers (39), and another study in Cambodia explained a link to forest-goers and multidrug-resistant malaria due to delayed care (40). In contrast, merchants or community leaders (included in the “other” category) may have more resources and social networks to facilitate clinic visits. These results suggest that malaria messaging and services should be specifically targeted at rural farm communities, for example, by collaborating with farm owners to provide on-site testing or flexible clinic hours (41, 42), thereby reducing the care-seeking gap.

Educational level showed a more complex relationship. Paradoxically, in Thailand, patients with pre-school or primary school education level had longer delays than those with no formal education. This counterintuitive result may reflect complex confounding: for instance, partially educated individuals initially self-medicate, feeling they recognize symptoms and only go to the clinic if the fever persists, whereas completely uneducated patients might immediately treat any fever as serious. Similar nuanced patterns have been observed in other settings; for example, basic literacy does not guarantee treatment adherence (43) or early reporting of cancer symptoms (44), suggesting that health literacy and cultural context may be more important than formal schooling.

Prior personal experience with malaria was associated with faster care-seeking: Thai patients who had malaria in the past 12 months sought care sooner than those without prior malaria episodes. Conversely, those who did not remember their previous malaria status delayed care in both countries. These patterns likely reflect experience and familiarity. People who have had malaria before may recognize the symptoms quickly and know where to go for treatment, whereas those without recent episodes may lack awareness of symptoms or the urgency of care (45). Forgetting whether one had malaria suggests a lack of engagement with malaria prevention, which could translate to delayed action. These findings highlight personal experience as a critical driver of timely care-seeking. Educating individuals with no prior malaria exposure, or those who do not recall past episodes, to recognize febrile symptoms and about the importance of early treatment is essential for improving response times. This was proven effective by a study conducted in Nigeria, which demonstrated the effectiveness of health education in enhancing caregivers’ knowledge of malaria and increasing their likelihood of seeking antimalarial treatment for febrile children (46).

Frequent bed net usage correlated strongly with quicker care-seeking in both countries. In Myanmar, daily net users had a shorter delay than non-users, and in Thailand, “some days” and “every day” users had faster care-seeking, respectively. This likely reflects that bed net use is a marker of malaria awareness and preventive behavior. Communities that receive insecticide-treated bed nets are often provided with accompanying health education, which has been associated with higher bed net ownership and improved health-seeking behaviors (47). Thus, bed net usage appears to be part of a broader “engaged” health profile, linking prevention and prompt treatment.

Interestingly, malaria-positive patients tended to present later than malaria-negative ones. Compared to malaria-negative patients, in Myanmar, *P. falciparum* patients delayed 10% longer and *P. vivax* 23% longer; in Thailand, *P. falciparum* delays were 27% longer, *P. viva*x 20% longer, and other malaria, such as *P. knowlesi*, *P. malariae*, and mixed infections, 32% longer. A plausible explanation is related to immunity and symptom severity. In endemic areas, people often acquire partial immunity: they can harbor parasites but experience milder or slower-onset symptoms (48). Thus, individuals who are infected, especially with *P. vivax*, which sometimes causes gradual symptom development (49), might not feel severely ill at first and delay care, whereas someone with a non-malarial fever (e.g., influenza) might feel acutely sick and seek help immediately. WHO notes that initial malaria symptoms can be “mild and difficult to recognize”, supporting this notion (50). In sum, our findings suggest that positive cases, despite being the true target, paradoxically present later, potentially due to attenuated early symptoms from partial immunity. This highlights the need to promote early diagnosis and timely treatment, even when symptoms such as mild fever may not appear serious, in endemic areas.

Seasonality emerged as a significant determinant of malaria care-seeking delay in both Myanmar and Thailand, though with contrasting patterns reflecting their distinct climatic contexts. In Myanmar, patients sought care more promptly during the peak rainy season in the third (July-September) and fourth (October-December) quarters compared to the dry season (January-March). This finding aligns with existing evidence indicating heightened malaria transmission during the rainy months in Myanmar, where increased mosquito density and parasite proliferation elevate community awareness of malaria symptoms and urgency for care (51, 52). In contrast, Thailand exhibited a different seasonal pattern, with delays in malaria care-seeking slightly increasing in the third and fourth quarters compared to the first quarter. This nuanced seasonal pattern likely reflects Thailand’s relatively consistent year-round malaria risk, particularly in border regions and forested areas, where perennial rainfall maintains suitable breeding habitats for mosquitoes (53). Additionally, prolonged exposure to persistent malaria risk may contribute to fatigue or complacency in seeking prompt care, as persistent low-level exposure could normalize fever episodes, reducing perceived urgency. These findings underline the necessity for country-specific and tailored approaches, sensitive to the local climatic and epidemiological contexts (54).

Unlike hypothetical assessments of health-seeking behavior that assess self-reported care-seeking intentions, the data from this study capture observed care-seeking behavior based on the duration of fever before facility attendance. This distinction enhances the validity of our findings, as they reflect observed behaviors rather than stated intentions, which are often subject to social desirability or optimistic assumptions (55). Furthermore, this study employed an AFT model, which captures aspects of care-seeking behavior that binary classifications of “timely” versus “delayed” care may overlook. By modeling the actual time from fever onset to diagnosis/treatment, the AFT approach reflects the full distribution of treatment delays and allows for a more nuanced understanding of how demographic, geographic, and behavioral factors influence the speed of care-seeking. This approach avoids relying on potentially arbitrary time thresholds and enables the identification of factors associated with shorter or longer delays across the entire time continuum, thereby supporting the design of more precisely targeted interventions. The findings from this study span two ethnically distinct settings in Myanmar and two malaria-endemic border regions in Thailand: one in the south, where *P. knowlesi* predominates, and Tha Song Yang District in the north, where *P. vivax* is more prevalent. In this context, tailored and regionspecific strategies are urgently needed to reinforce a comprehensive health-seeking mindset that accelerates malaria elimination in both Myanmar and Thailand.

### Limitations

Several limitations warrant consideration. First, fever onset was self-reported, introducing potential recall bias. Patients may have misremembered or overlooked early symptoms, leading to under- or overestimation of care-seeking delays. Second, the study population comprised only individuals who sought care at malaria clinics, thereby excluding those who delayed indefinitely or relied on informal care. Consequently, our findings reflect delays among individuals who eventually sought care and may therefore underestimate the true extent of delays in the broader community. Unmeasured confounders such as travel distance to clinics or perceived severity of illness may also have influenced care-seeking behavior, but were not captured. Further research using qualitative approaches, such as in-depth interviews or focus group discussions, would help elucidate the contextual and behavioral drivers of delayed malaria care-seeking. Some categorical variables, such as “other” occupations or ethnicities, combined heterogeneous groups, potentially masking meaningful intra-group variation. Moreover, we used secondary data for analysis, where fever duration was recorded in whole days rather than hours, reducing temporal precision. This precluded accurate assessment of adherence to the WHO-recommended 24-hour window for malaria diagnosis and treatment. Despite these limitations, the study’s large sample size, multi-country design, and standardized data collection strengthen the validity of its findings and support the observed patterns in care-seeking behavior.

## Conclusions

This study highlights delays in malaria care-seeking among febrile patients attending community-based malaria facilities in Myanmar and Thailand. Using longitudinal data and survival analysis, we identified several key risk factors associated with longer time to care-seeking, including ethnic minority status, agricultural occupation, uncertain malaria history, lower education, and infection with *Plasmodium* species. In contrast, consistent use of bed nets and a history of recent malaria were associated with shorter delays. These findings underscore the importance of addressing both structural and behavioral determinants of health in malaria-endemic settings. Tailored strategies that engage high-risk populations, particularly ethnic minorities and agricultural workers, and reinforce the benefits of early diagnosis and prevention may enhance timely care-seeking. Strengthening health education, community engagement, and access to malaria services remain essential to accelerate malaria elimination efforts across the GMS.

## Figures and Tables

**Table 1:** Fever duration among patients attending community-based malaria facilities in Myanmar and Thailand

	n	Mean fever days (SD)	Median fever days (range)
**Myanmar**	**2960**	**1.8 (1.3)**	**1 (1–14)**
Banmauk	997	2.1 (1.1)	2 (1–12)
Paletwa	1963	1.7 (1.4)	1 (1–14)
**Thailand**	**15576**	**2.9 (1.8)**	**2 (1–45)**
Bannang Sata	897	3.2 (1.6)	3 (1–36)
Saba Yoi	688	4.6 (2.5)	5 (1–30)
Tha Song Yang	13991	2.8 (1.7)	2 (1–45)

**Table 2:** Factors associated with time to malaria care-seeking in Myanmar (n = 2,960)

Characteristic	n (%)^a^	Time ratio (95% CI)	*p*-value
**Age group** (years)			
< 5	454 (15.34)	Reference	
5–14	690 (23.31)	1.05 (0.99, 1.11)	0.109
15–34	948 (32.03)	0.99 (0.91, 1.09)	0.901
≥ 35	868 (29.32)	1.05 (0.95, 1.15)	0.353
**Sex**			
Female	1186 (40.07)	Reference	
Male	1774 (59.93)	1.01 (0.98, 1.05)	0.467
**Ethnicity**			
Chin	1339 (45.24)	Reference	
Rakhine	578 (19.53)	0.99 (0.94, 1.04)	0.605
Shan	969 (32.74)	1.48 (1.41, 1.55)	<0.001
Other	74 (2.50)	1.24 (1.08, 1.41)	0.002
**Occupation**			
Unemployed	87 (2.94)	Reference	
Agriculture/Farmer	1369 (46.25)	1.12 (1.00, 1.25)	0.042
Child/Student	1271 (42.94)	0.96 (0.84, 1.09)	0.511
Mining	111 (3.75)	1.15 (1.00, 1.34)	0.058
Other (Merchant, Monk, etc.)	122 (4.12)	0.98 (0.84, 1.14)	0.774
**Education**			
No formal education	426 (14.39)	Reference	
Pre/Primary school	2165 (73.14)	1.02 (0.96, 1.08)	0.518
Secondary school	326 (11.01)	1.03 (0.95, 1.12)	0.438
University or higher	43 (1.45)	0.94 (0.80, 1.12)	0.500
**Malaria history within past 12 months**			
No	2779 (93.89)	Reference	
Yes	137 (4.63)	1.05 (0.96, 1.15)	0.290
Do not remember	44 (1.49)	1.26 (1.09, 1.47)	0.002
**Bed net usage**			
Not at all	340 (11.49)	Reference	
Some days	159 (5.37)	1.07 (0.97, 1.18)	0.199
Every day	2461 (83.14)	0.93 (0.88, 0.99)	0.023
**Malaria diagnosis**			
Negative	2563 (86.59)	Reference	
*P. falciparum*	208 (7.03)	1.10 (1.02, 1.19)	0.010
*P. vivax*	174 (5.88)	1.23 (1.13, 1.34)	<0.001
Other malaria (mixed species)	15 (0.51)	1.01 (0.76, 1.34)	0.963
**Diagnosis period**			
Q1 (January-March)	462 (15.61)	Reference	
Q2 (April-June)	541 (18.28)	0.99 (0.92, 1.05)	0.690
Q3 (July-September)	1078 (36.42)	0.91 (0.86, 0.96)	0.001
Q4 (October-December)	879 (29.70)	0.92 (0.87, 0.98)	0.005

a Rounding may cause percentages to sum slightly more or less than 100%.

**Table 3:** Factors associated with time to malaria care-seeking in Thailand (n = 15,576)

Characteristic	n (%)^a^	Time ratio (95% CI)	*p*-value
**Age group** (years)			
< 5	1819 (11.68)	Reference	
5–14	4721 (30.31)	0.98 (0.96, 1.01)	0.243
15–34	4681 (30.05)	1.03 (0.98, 1.08)	0.202
≥ 35	4355 (27.96)	1.03 (0.98, 1.08)	0.210
**Sex**			
Female	6554 (42.08)	Reference	
Male	9022 (57.92)	1.00 (0.98, 1.01)	0.579
**Ethnicity**			
Thai	3985 (25.58)	Reference	
Karen	11,494 (73.79)	0.98 (0.96, 1.00)	0.067
Other	97 (0.62)	1.03 (0.93, 1.14)	0.550
**Occupation**			
Unemployed	580 (3.72)	Reference	
Agriculture/Farmer	4669 (29.98)	1.07 (1.02, 1.11)	0.002
Child/Student	7125 (45.74)	0.98 (0.93, 1.04)	0.512
Other (Merchant, Monk, etc.)	3202 (20.56)	0.94 (0.90, 0.98)	0.003
**Education**			
No formal education	6186 (39.71)	Reference	
Pre/Primary school	6671 (42.83)	1.02 (1.00, 1.04)	0.026
Secondary school	2419 (15.53)	1.01 (0.98, 1.04)	0.478
University or higher	300 (1.93)	1.04 (0.98, 1.10)	0.222
**Malaria history within past 12 months**			
No	14,488 (93.01)	Reference	
Yes	516 (3.31)	0.89 (0.86, 0.93)	<0.001
Do not remember	572 (3.67)	1.10 (1.06, 1.14)	<0.001
**Bed net usage**			
Not at all	1319 (8.47)	Reference	
Some days	3457 (22.19)	0.89 (0.86, 0.92)	<0.001
Every day	10,800 (69.34)	0.83 (0.80, 0.85)	<0.001
**Malaria diagnosis**			
Negative	13,927 (89.41)	Reference	
*P. falciparum*	43 (0.28)	1.27 (1.09, 1.46)	0.002
*P. vivax*	1569 (10.07)	1.20 (1.17, 1.23)	<0.001
Other malaria^b^	37 (0.24)	1.32 (1.12, 1.56)	<0.001
**Diagnosis period**			
Q1 (January-March)	2773 (17.80)	Reference	
Q2 (April-June)	3950 (25.36)	0.99 (0.96, 1.01)	0.282
Q3 (July-September)	5285 (33.93)	1.03 (1.00, 1.05)	0.017
Q4 (October-December)	3568 (22.91)	1.06 (1.04, 1.09)	<0.001

a Rounding may cause percentages to sum slightly more or less than 100%.

b Other malaria included mixed infections, *P. knowlesi*, *P. malariae*, and *P. ovale* infections.

## Data Availability

All data generated or analyzed during this study are included in the article. The de-identified raw dataset is available from the corresponding author upon reasonable request.

## Abbreviations

ACT: artemisinin-based combination therapy
AFT: accelerated failure time
AIC: Akaike Information Criterion
BIC: Bayesian Information Criterion
CI: confidence interval
CRF: case record form
GMS: Greater Mekong Subregion
ICMV: integrated community malaria volunteer
MC: malaria clinic
MHV: migrant health volunteer
NGO: non-governmental organization
RDT: rapid diagnostic test
SD: standard deviation
TR: time ratio
VHV: village health volunteer
VIF: variance inflation factor
WHO: World Health Organization
